# Deep learning based atomic defect detection framework for two-dimensional materials

**DOI:** 10.1038/s41597-023-02004-6

**Published:** 2023-02-14

**Authors:** Fu-Xiang Rikudo Chen, Chia-Yu Lin, Hui-Ying Siao, Cheng-Yuan Jian, Yong-Cheng Yang, Chun-Liang Lin

**Affiliations:** 1 Department of Electrophysics, National Yang Ming Chiao Tung University, Hsinchu City, Taiwan; 2 Department of Computer Science and Information Engineering, National Central University, Taoyuan City, Taiwan; 3 Department of Electrical and Computer Engineering, University of California, Davis, CA USA; 4 Department of Computer Science and Engineering, Yuan Ze University, Taoyuan City, Taiwan

**Keywords:** Electronic devices, Electrical and electronic engineering

## Abstract

Defects to popular two-dimensional (2D) transition metal dichalcogenides (TMDs) seriously lower the efficiency of field-effect transistor (FET) and depress the development of 2D materials. These atomic defects are mainly identified and researched by scanning tunneling microscope (STM) because it can provide precise measurement without harming the samples. The long analysis time of STM for locating defects in images has been solved by combining feature detection with convolutional neural networks (CNN). However, the low signal-noise ratio, insufficient data, and a large amount of TMDs members make the automatic defect detection system hard to be applied. In this study, we propose a deep learning-based atomic defect detection framework (DL-ADD) to efficiently detect atomic defects in molybdenum disulfide (MoS_2_) and generalize the model for defect detection in other TMD materials. We design DL-ADD with data augmentation, color preprocessing, noise filtering, and a detection model to improve detection quality. The DL-ADD provides precise detection in MoS_2_ (F2-scores is 0.86 on average) and good generality to WS_2_ (F2-scores is 0.89 on average).

## Background & Summary

Transition metal dichalcogenides (TMDs), as two-dimensional (2D) materials, have been predicted to have huge application potential in the solar energy and semi-conductor industry^1–5^. Nevertheless,TMDs usually obtain many defects because of the low formation energy of chalcogen vacancies^6^. When attempting to construct a high-performance field-effect transistor, defects can affect contact resistance^7^, result in Fermi-level pinning^7,8^, and reduce carrier mobility^9,10^.

To prevent defects from affecting the performance of TMD devices, researchers usually use optical methods^11,12^, probing techniques^13–16^ and transmission electron techniques^17,18^ to measure the defect distribution on each TMD surface. Optical methods have advantages, such as large area and element detection, especially excellent in X-ray photoelectron spectroscopy measurement^19^. However, the spatial resolution of optical methods cannot exceed the diffraction limit of approximately 0.1 µm and can sometimes harm the sample because of heat generated from pumping light. The problem can be conquered by applying probing techniques, particularly scanning tunneling microscopy (STM) which can provide ultra-high-resolution images without harming the sample. Because of the extremely high resolution of the image, STM measurements exhibit zero deviations and element recognition in defect density estimation. Yet, the STM measurements require scanning hundreds of images and counting thousands of defects from images one by one, which takes a lot of time. Therefore, estimating the defect density of samples by STM will require roughly dozens hours for counting defects. Artificial intelligence techniques are used to solve this problem.

Deep learning-based STM analysis provides substantial advantages but also presents problems when applying sensing defect variance in TMDs-based field-effect transistor (FET). Based on many training images, most studies have achieved high accuracy in detecting defects^20–22^. They used 3500 to 7500 images in their reports, depending on the signal-to-noise ratio. Using STM to collect this amount of data must take months. Furthermore, the STM tip is frequently exposed to residual chemical and poor conducting areas, while scanning FETs, resulting in images with highly unstable quality and low signal-to-noise ratio. Density functional theory (DFT) calculation is used to generate a large amount of mimic data to solve this problem^23^. However, inadequate and low-quality experiment data can still result in model overfitting and showing low accuracy.

Furthermore, an increasing number of materials that can be applied to FET will require defect detection. A well-trained model must be retrained when applying to different materials^22^ because the appearance of defects are changed, resulting in a large data collection cost and model training time. The combination of these problems makes detecting defects in TMD-based FETs difficult.

In this study, we propose a deep learning-based atomic defect detection framework (DL-ADD) for diagnosing defects in TMD-based FETs to address the problem of insufficient and low-quality data and to improve model generality. In DL-ADD, we design a data augmentation module, a color preprocessing module, a noise filtering module, and a detection model to reduce analysis time and improve detection accuracy. We also develop a data augmentation module to generate more pseudo data for the training model to depress overfitting. Color preprocessing, and noise filtering modules are designed to improve the quality of data. We implement U-Net^24^ as the detection model to accurately locate and identify the atomic defect because it can segment images faster and more precisely. Furthermore, we conduct experiments based on molybdenum disulfide (MoS_2_) and tungsten disulfide (WS_2_) images to evaluate the accuracy and the generality of DL-ADD. The defects are defined as impurities^8,25^ and voids^13,26^ since these two defects are frequently exist in MoS_2_ and WS_2_. The proposed achieves an F1-score of 0.89 for impurities and 0.80 for voids in MoS_2_ with an extremely small amount of data (approximately 70 images). We use the MoS_2_ model to differentiate the WS_2_ defects without retraining. The F1-score for voids in WS_2_ is 0.94, that is, the proposed DL-ADD can be effectively used for TMD-based FETs with limited and low-quality data and widely applied to other 2D materials.

## Methods

**Fig. 1:**
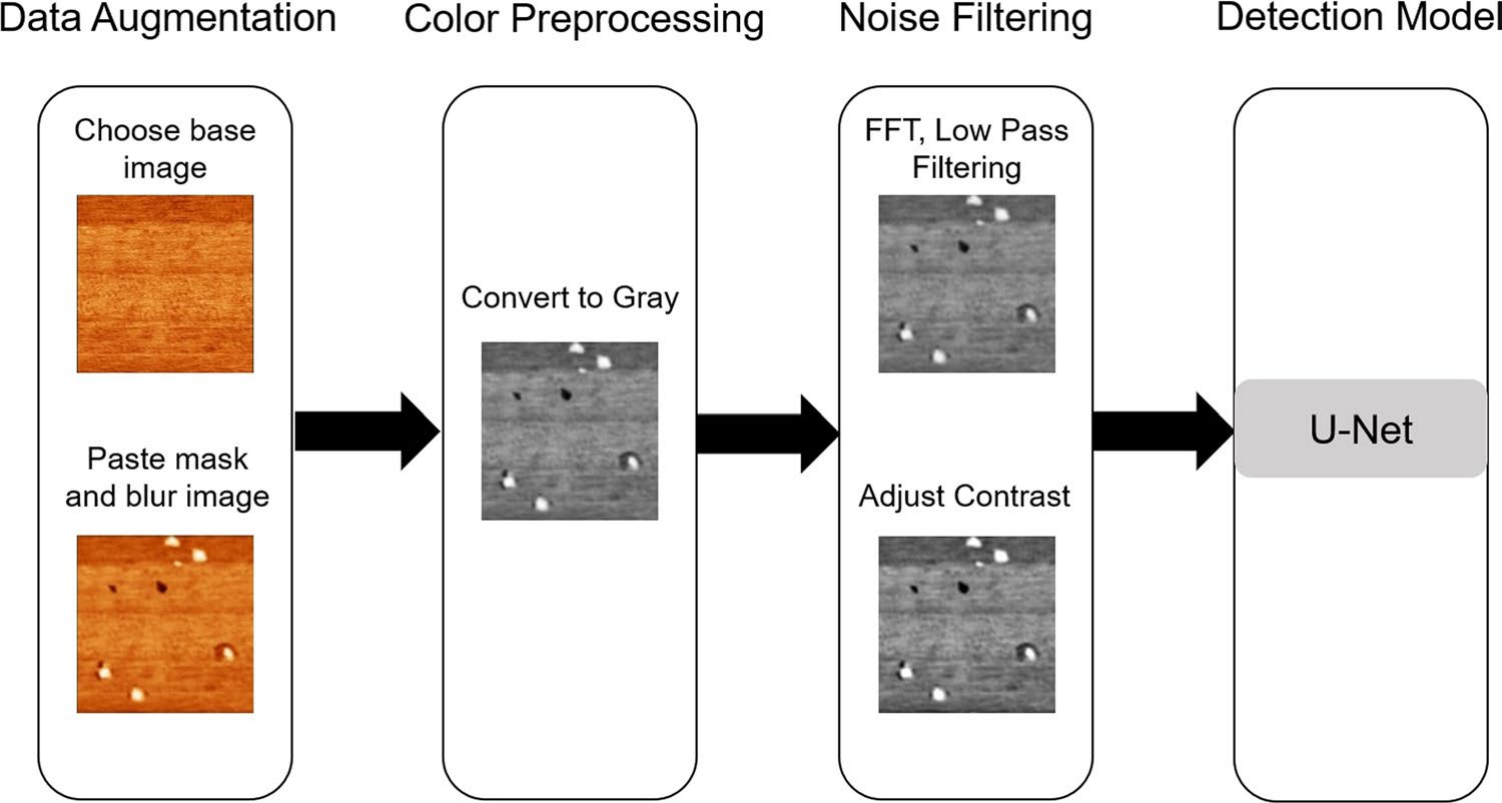
DL-ADD Framework.

As shown in Fig. 1, we propose a deep learning-based atomic defect detection framework (DL-ADD) for 2D materials. Three data preprocessing modules and a detection model were included. The code of DL-ADD is accessible at GitHub: https://github.com/MeatYuan/MOS2.

### Experimental detail

This research applies room temperature STM (RT-STM) to scan MoS_2_ and WS_2_ surfaces at vapor pressure 1 × 10^−10^ torr. Single crystal WS_2_ and MoS_2_ bulk were both bought from Structure Probe Inc. The WS_2_ and MoS_2_ crystals were applied with the different processes before scanned to increase the number of impurities and voids. The MoS_2_ sample was kept at normal pressure after cleaved by mechanical exfoliation and were transferred to make field effect transistor. The WS_2_ sample was cleaved at an ultra-high vacuum chamber and heated for 200 for 12 hours to increase only the number of voids. All images are scanned in constant current mode with −1 V sample bias and 1 nA tunneling current. By the help of this method, statistical property of defect density was researched^16^.

### Data augmentation

**Fig. 2:**
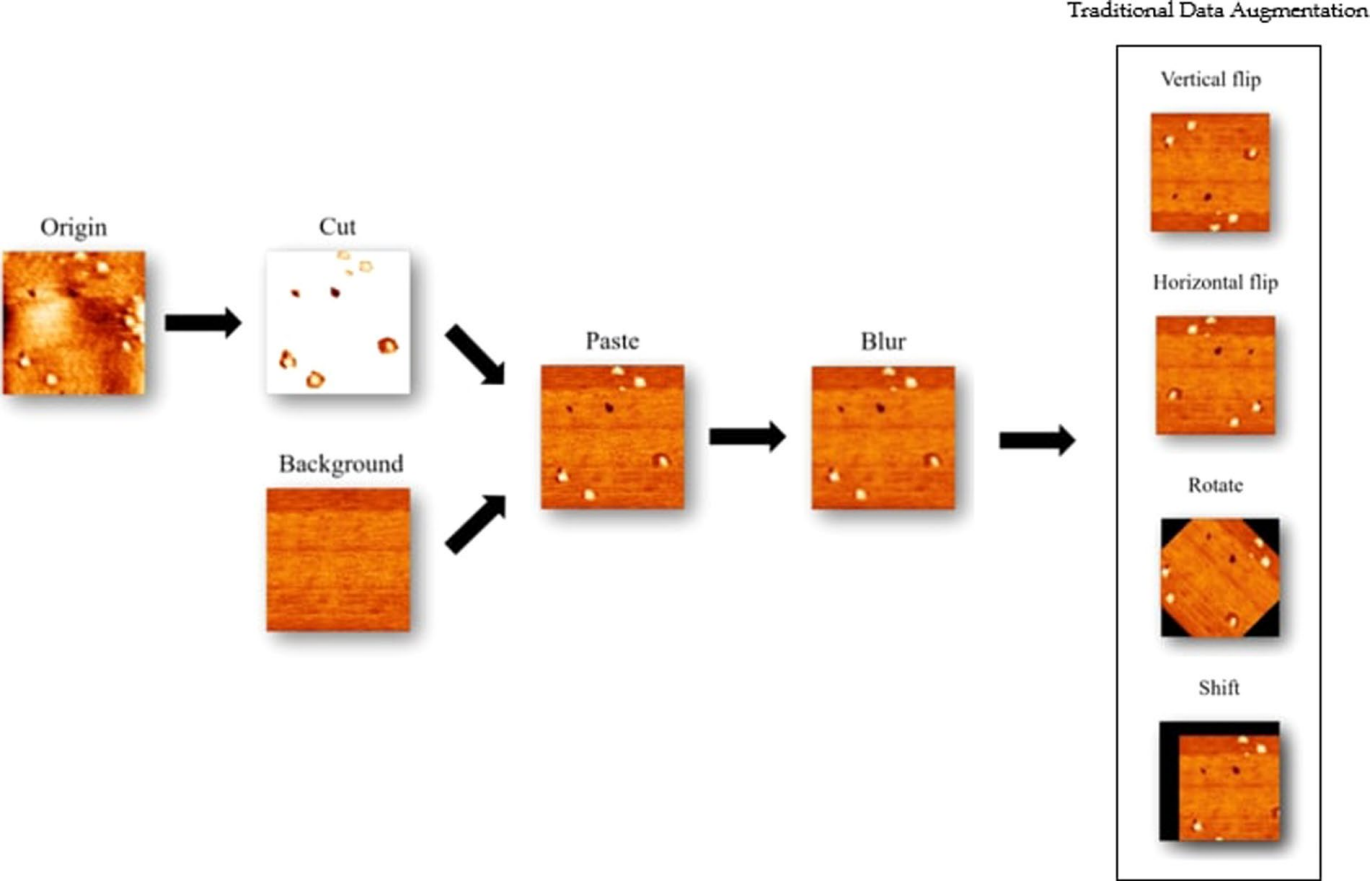
Data Augmentation Process.

To train an accurate model, a certain amount of data is required. However, collecting 2D material images requires much labor and takes a long time. Therefore, we designed a data augmentation process to increase the amount of training data. The process of data augmentation is shown in Fig. 2. To augment data and maintain the diversity of data, we first manually create the defect data and then augment more data using traditional augmentation methods. We cut the voids and impurities from the training data. After that, we selected a clear background of the MoS_2_ surface and pasted the cut defects into the background images. The proportions of impurities and voids are randomized to simulate real images. We used the Gaussian blur on images to reduce the discontinuity of the edge to make the boundary of the cut images and background smoother. Finally, we randomly choose horizontal flip, vertical flip, rotation, and shift methods to augment data.

### Color preprocessing

Compared to color images, the gray images are easier to remove noise and reduce data difference. Therefore, we resize images to 256 × 256 pixels and convert them to gray in this module.

### Noise filtering

In scanning probe microscope research, the quality of experiment data strongly depends on tip conditions. When dealing with a rough sample (root mean square roughness above 1 nm), a tip can easily hit the sample, resulting in a large amount of data scanned in bad conditions. Under this condition, high-frequency noise shows stronger intensity than signals on the image, making it more difficult for the model to detect defects. We first use Fast Fourier Transform (FFT) on the images, then a low pass filter to remove high-frequency signals before performing inverse FFT. Finally, we adjust the images contrast higher to make the impurity and void more visible.

### Detection model

**Fig. 3:**
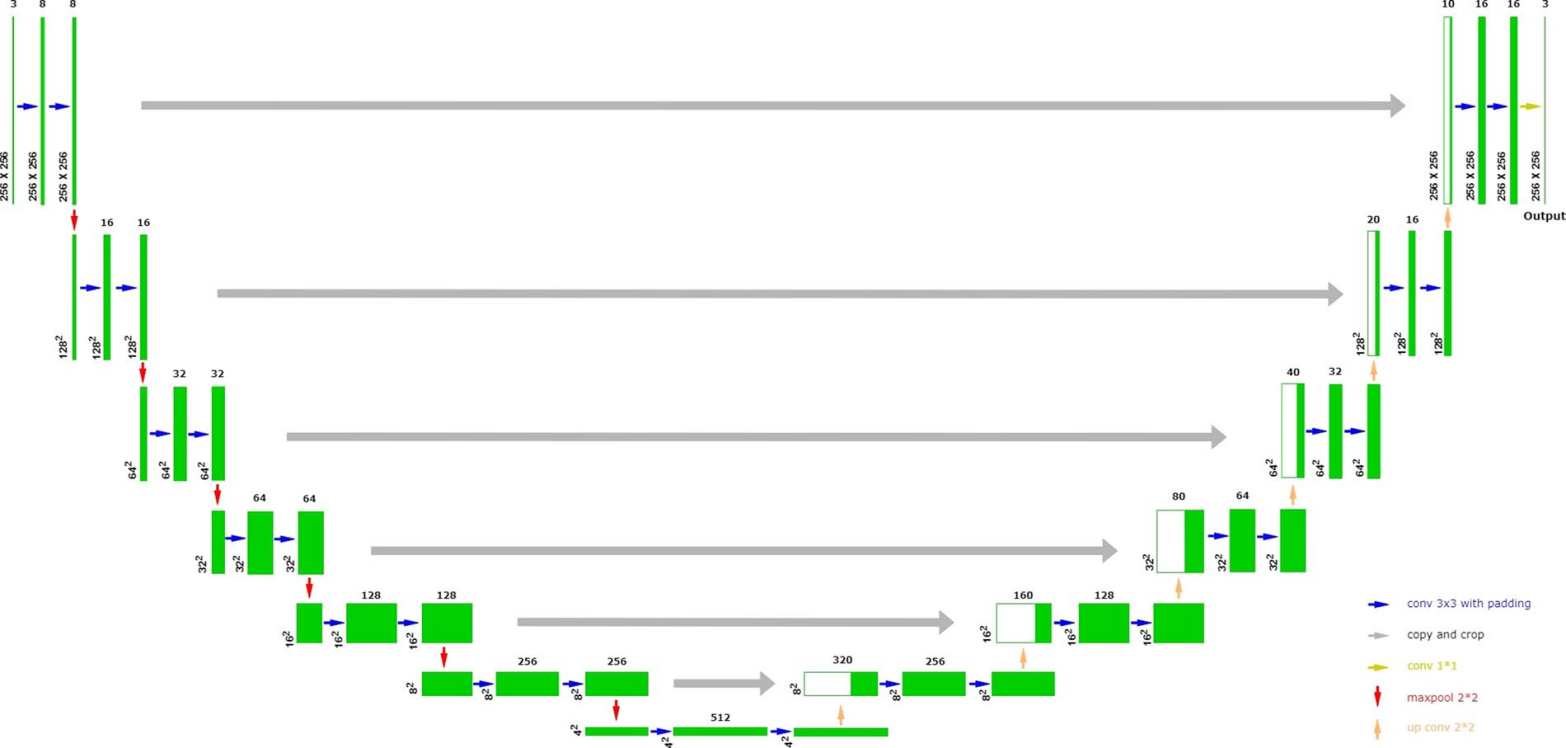
Model architecture.

We choose U-Net as the defect detection model since it can be trained with few images and achieve precise segmentation. U-Net^24^ is a convolutional network architecture that consists of a contracting path for downsampling and an expansive path for upsampling, as shown in Fig. 3. Six identical parts are used in the downsampling, each comprising two convolutional layers with a 3 × 3 kernel size and one max pooling layer. Also six identical parts are used in the upsampling, each comprising two convolutional layers with a 3 × 3 kernel size, one transposed convolution layer, and a concatenate with the same size as the previous feature map. Finally, we use three convolutional layers to output a 256 × 256 size image.

## Data Records

The actual size of MoS_2_ and WS_2_ images are separately 50 × 50 *nm*^2^ and 60 × 60 *nm*^2^ stored in Bitmap format (BMP) with 256 pixels, which is the most suitable size to distinguish and detect those defects. All of the data is kept at Open Science Framework at OSF^27^: 10.17605/OSF.IO/ZXGTJ

## Technical Validation

In this section, we will evaluate DL-ADD. We first compare DL-ADD and YOLOv4^28^ and then conduct ablation studies of image preprocessing module and data augmentation module of DL-ADD. Furthermore, to demonstrate the generality of DL-ADD, we apply it to WS_2_ material which also contains voids and impurities. A void can be labeled as a black dip appears on the surface, while an impurity can be labeled as bright protrusion. We scanned the entire scannable area in one MoS_2_ FET sample and obtained 90 images to evaluate DL-ADD in the following experiments. Seventy images are used as training dataset while twenty images are used as testing dataset.

The evaluation metrics are recall, precision, F1-score and F2-score. False Positive (FP) is referred to as pseudo defects. False Negative (FN) is the defects that the model does not detect. True Positive (TP) is the true defects that are correctly classified. F-Score is the harmonic average of precision and recall. Since the defect is more important, we add F2-score in the following result.

1 $$Recall=\frac{TP}{TP\,+\,FN}$$

2 $$Precision\,Rate=\frac{TP}{TP\,+\,FP}$$

3 $${F}_{\beta }=(1\,+\,{\beta }^{2})\frac{Precision\ast Recall}{({\beta }^{2}\ast Precision)\,+\,Recall}$$

### Model evaluation

**Fig. 4:**
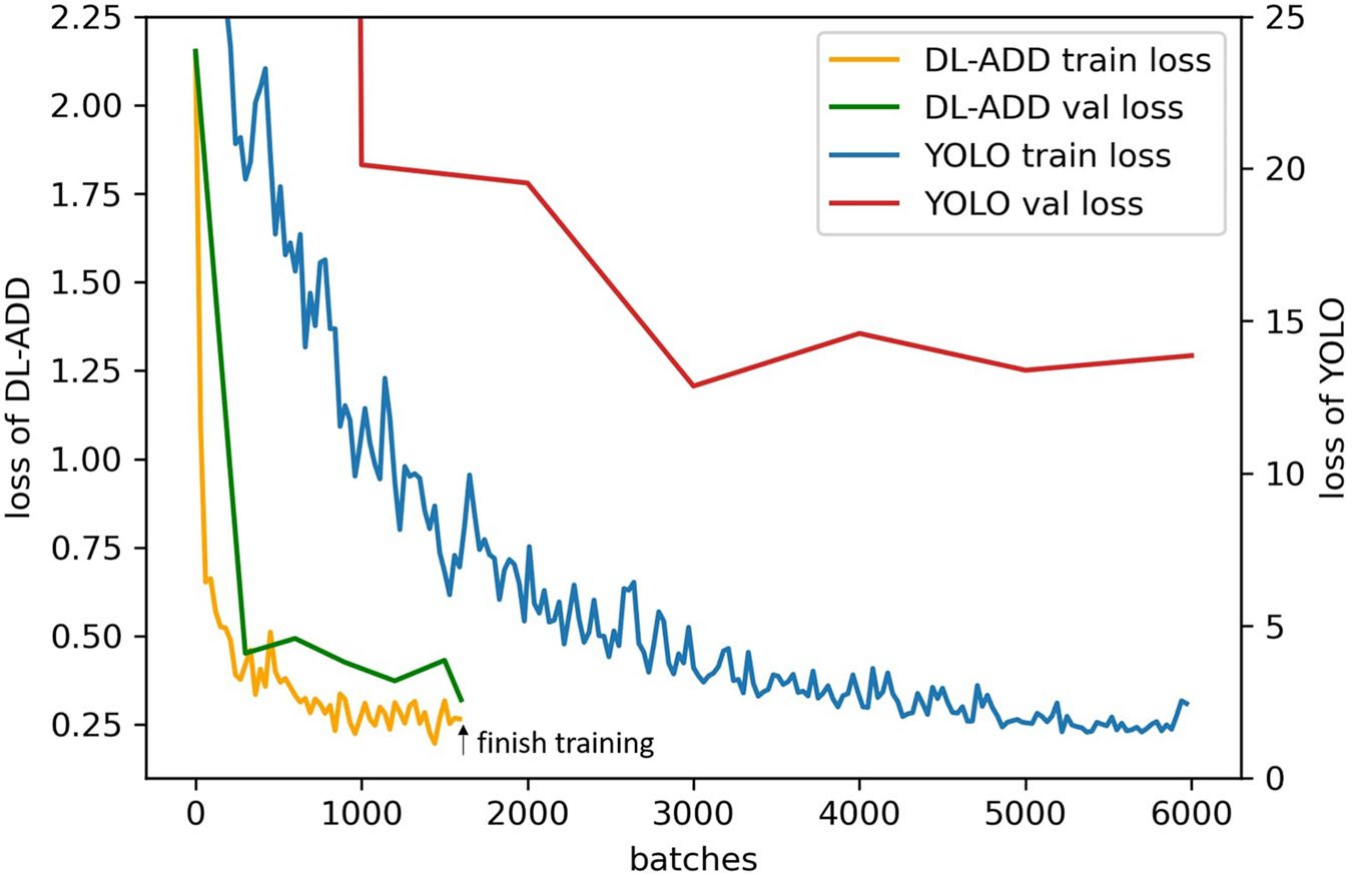
Training and validation loss of models. DL-ADD ends training at 1600 batches.

**Fig. 5:**
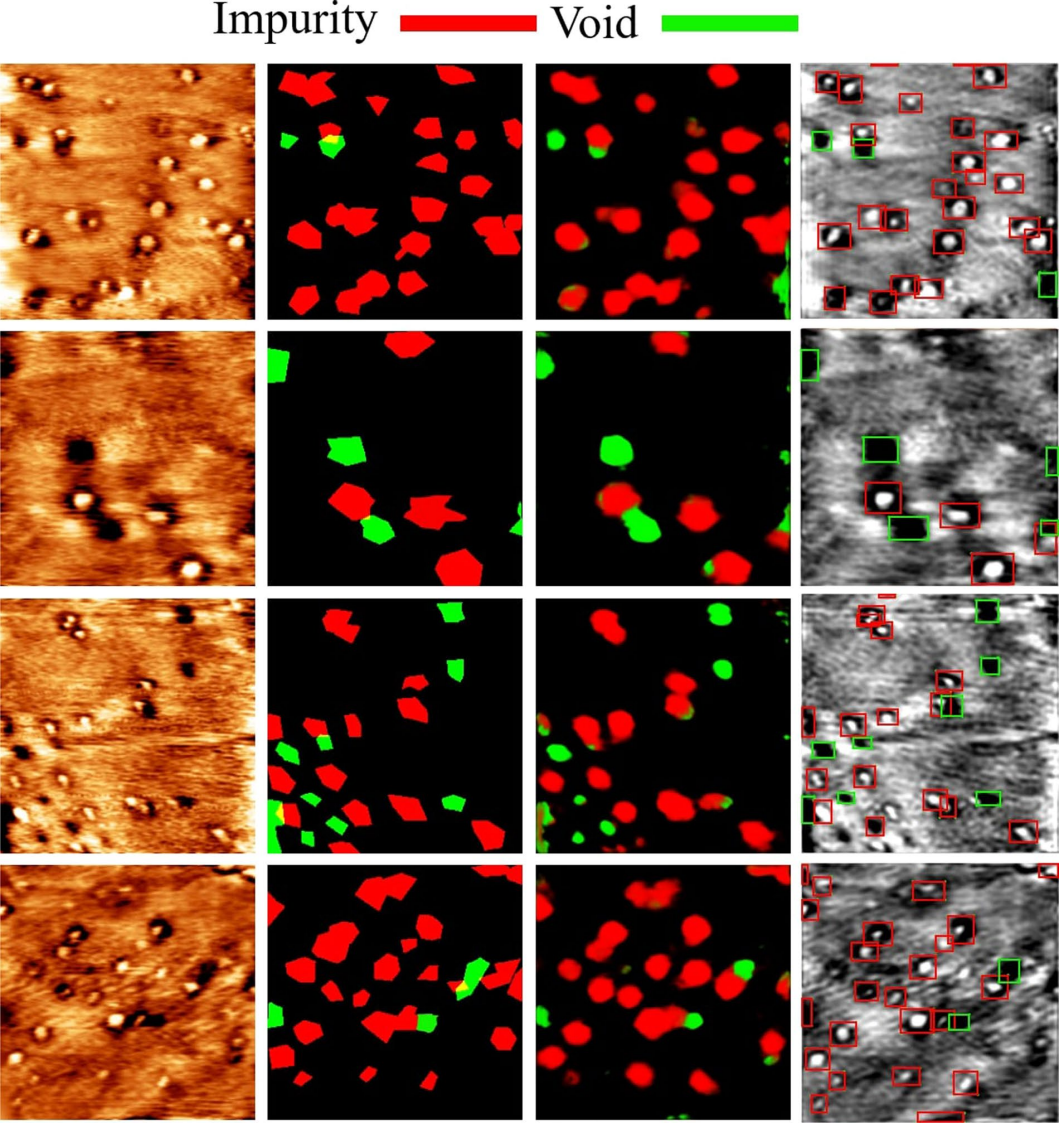
Result of MoS_2_ defect detection. STM MoS_2_ image (left), Ground Truth (middle left), DL-ADD detection (middle right), YOLOV4 detection (right). The size of the scanning frame for each image is 50 × 50 nm^2^.

Table 1 compares DL-ADD with YOLOv4 to evaluate the model, indicating that F1-score of DL-ADD outperforms the YOLO model in terms of voids and impurities. As shown in Fig. 4, the loss of DL-ADD drops faster and more dramatically compared to YOLOv4. The difference of loss clearly indicates YOLOv4 is more difficult to converge. This may be due to the complexity of YOLOv4 increasing the necessity of data numbers. As such, the simple structure of the U-Net of DL-ADD can achieve better performance based on little data. Some of the DL-ADD and YOLO results are shown in Fig. 5.

**Table 1:** The accuracy of DL-ADD and YOLOv4 models.

	DL-ADD		YOLOv4	
	Impurity	Void	Impurity	Void
Recall	0.90	0.82	0.76	0.61
Precision	0.95	0.77	0.85	0.65
F1-Score	0.92	0.80	0.89	0.70
F2-Score	0.91	0.81	0.92	0.73

### Data augmentation validation

In DL-ADD, we use data augmentation by manually imitating data. We validate the effect of data augmentation in Table 2. Both defects are detected more accurately after the process is used. The augmentation process primarily balance model precision and recall relief the miss-alarm problem.

**Table 2:** The detection accuracy of DL-ADD model with and without data augmentation.

	Origin		Augmentation	
	Impurity	Void	Impurity	Void
Recall	0.77	0.92	0.90	0.82
Precision	0.98	0.57	0.95	0.77
F1-Score	0.86	0.70	0.92	0.80
F2-Score	0.80	0.82	0.91	0.81

### Noise filtering validation

The noise signals mainly exist in the high-frequency region, while the defect signals are mostly found in the low-frequency region. Thus, we design a low pass filter in DL-ADD to improve the quality of images. We show the effects of noise filtering methods in Table 3. The results show that our procedure successfully improves the signal-to-noise ratio and significantly improves accuracy regardless of recall or precision.

**Table 3:** The detection accuracy of DL-ADD with and without preprocessing.

	Origin		Preprocess	
	Impurity	Void	Impurity	Void
Recall	0.49	0.88	0.77	0.92
Precision	0.94	0.33	0.98	0.57
F1-Score	0.64	0.48	0.86	0.70
F2-Score	0.54	0.66	0.80	0.82

### Generality validation

**Fig. 6:**
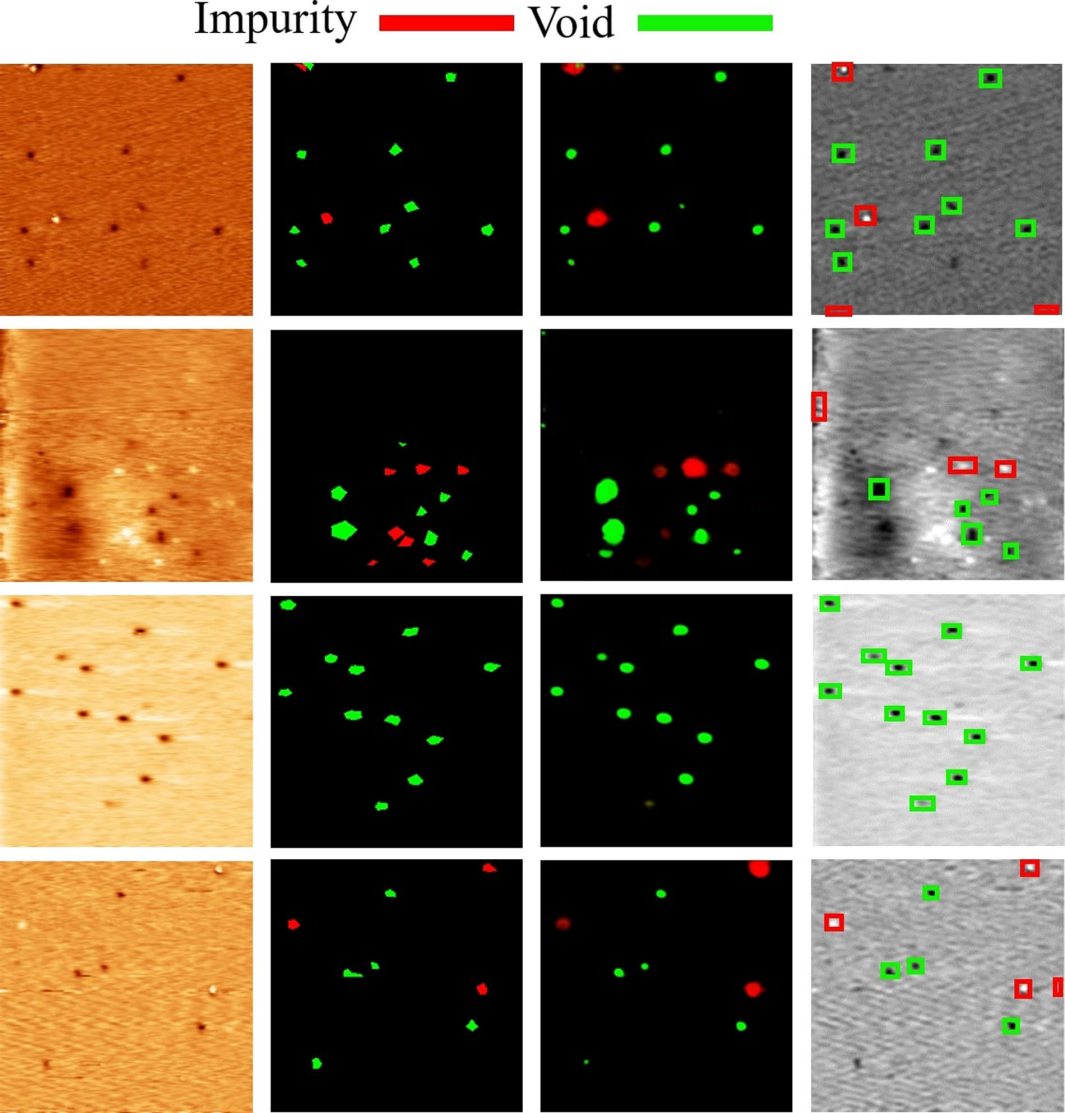
Result of WS_2_ defect detection. STM WS_2_ image (left), Ground Truth (middle left), DL-ADD detection (middle right), YOLOv4 detection (right).The size of the scanning frame for each image is 60 × 60 nm^2^.

Some 2D materials are similar. If DL-ADD can be generalized to these materials, we can save much model training time. We apply DL-ADD which is trained with MoS_2_ to detect the defects of WS_2_. The results of detecting defects on 15 WS_2_ images are shown in Fig. 6 and Table 4. The F1-Score and F2-Score of DL-ADD are significantly higher than those of YOLO. The impurity data of the two training models in the MoS_2_ report are barely different, but the difference of void data is more obvious. Among the two materials, the accuracy of DL-ADD does not decrease too much, but the F1-Score of YOLO is less than 0.6, indicating that YOLO is completely overfitted on MoS_2_. That is, the generality of DL-ADD is better than YOLO.

**Table 4:** Generality validation to WS_2_ of each model.

	DL-ADD		YOLOv4	
	Impurity	Void	Impurity	Void
Recall	0.95	0.89	0.61	0.74
Precision	0.63	0.99	0.43	0.77
F1-Score	0.76	0.94	0.50	0.76
F2-Score	0.86	0.91	0.57	0.75

### Training and inference time

In addition to improving accuracy, the detection time is essential to defect detection. We compare the training and inference times of the two models, as shown in Table 5. We use the RTX 3090 to train two models. DL-ADD takes 29 minutes to finish model training, and YOLOv4 takes 91 minutes, more than three times that of DL-ADD. In the inference speed, DL-ADD takes 0.17 seconds per image, and YOLOv4 takes 0.15 seconds per image, and there is not much difference between the two.

**Table 5:** Training and inference time of each model.

	DL-ADD	YOLOv4
Training time (min)	29	91
Inference time (s)	0.17	0.15

## Usage Notes

In this study, we proposed a DL-ADD framework to efficiently detect atomic defects in TMD-based FET. In DL-ADD, we designed an image preprocessing module, a data augmentation module, and a detection model. With low quality and small amounts of data, DL-ADD performed well in detecting defects (F-score > 0.85 on average) and resisting intense noise, significantly lowering the restriction to apply this model for FET research. The good generality of DL-ADD further increased the convenience of this model, allowing to be used as a simple label tool while changing to other materials in the TMD family. Overall, DL-ADD demonstrated strong noise resistance, high accuracy, and good generality. This study lowered the threshold for using CNN models with the aforementioned properties and significantly improved the efficiency of the atomic defect detection process.

## Data Availability

All the code to produce the results of this paper is accessible at: https://github.com/MeatYuan/MOS2.We all use Python and jupyter notebook.
